# Decomposing Complexity Preferences for Music

**DOI:** 10.3389/fpsyg.2019.00674

**Published:** 2019-04-03

**Authors:** Yaǧmur Güçlütürk, Rob van Lier

**Affiliations:** ^1^Artificial Cognitive Systems Lab, Cognitive Artificial Intelligence Department, Donders Institute for Brain, Cognition and Behaviour, Radboud University, Nijmegen, Netherlands; ^2^Perception and Awareness Lab, Cognitive Psychology Department, Donders Institute for Brain, Cognition and Behaviour, Radboud University, Nijmegen, Netherlands

**Keywords:** complexity, liking, music, preferences, individual differences, cluster analysis

## Abstract

Recently, we demonstrated complexity as a major factor for explaining individual differences in visual preferences for abstract digital art. We have shown that participants could best be separated into two groups based on their liking ratings for abstract digital art comprising geometric patterns: one group with a preference for complex visual patterns and another group with a preference for simple visual patterns. In the present study, building up on these results, we extended our investigations for complexity preferences from highly controlled visual stimuli to ecologically valid stimuli in the auditory modality. Similar to visual preferences, we showed that music preferences are highly influenced by stimulus complexity. We demonstrated this by clustering a large number of participants based on their liking ratings for song excerpts from various musical genres. Our results show that, based on their liking ratings, participants can best be separated into two groups: one group with a preference for more complex songs and another group with a preference for simpler songs. Finally, we considered various demographic and personal characteristics to explore differences between the groups, and reported that at least for the current data set age and gender to be significant factors separating the two groups.

## 1. Introduction

Like its visual counterpart, music complexity has been shown to have an important role in determining whether a song will be liked or not (Heyduk, 1975; North and Hargreaves, 1995; Orr and Ohlsson, 2001; Marin and Leder, 2013; Marin et al., 2016). Distinct from the visual art objects, music has a temporal dimension. Therefore, its complexity can be defined differently than visual complexity. For example, borrowing ideas from Shannon's information theory^1^, songs that have highly predictable rhythm and/or melodies are considered simpler compared to those that have unpredictable rhythm or melodies.

However, defining music complexity remains difficult given its subjective nature (Shmulevich and Povel, 2000; Sallavanti et al., 2015) (for reviews on music perception and complexity see Streich, 2007; Stevens, 2012; Marin and Leder, 2013). Much like its visual counterpart; it is a multidimensional construct (Streich, 2007). Along with rhythm and melody predictability, subjective music complexity was shown to depend on several other factors. These dimensions or factors cover properties such as frequency and/or number of events, harmony and syncopation, variability, and number of instruments. Often the definitions of music complexity adopted in different studies have focused on only one of these properties, e.g., rhythm and meter complexity (Shmulevich and Povel, 2000; Thul and Toussaint, 2008; Vuust and Witek, 2014), instrumental complexity (Percino et al., 2014), tonal complexity (Weiss and Muller, 2015), and harmonic complexity (Marsik et al., 2014). Few other studies took an integrated approach by combining multiple music properties (Streich, 2007; Mauch and Levy, 2011; Marin and Leder, 2013).

Unfortunately, to the best of our knowledge, an objective measure of complexity of music which agrees very well with subjective complexity ratings does not exist. As discussed above, many studies investigated this question, but none have provided a measure comparable to e.g., the Kolmogorov complexity for visual stimulus [which for abstract visual patterns has been shown to correlate almost perfectly with subjective complexity; (Güçlütürk et al., 2016)]. Therefore, several studies relied on aggregating subjective complexity ratings provided by participants (Heyduk, 1975; Orr and Ohlsson, 2001, 2005; Rentfrow et al., 2011; Madison and Schiölde, 2017), while several other studies utilized computational measures of music complexity such as entropy related measures, e.g., Shannon's entropy, entropy rate, and excess entropy (Madsen and Widmer, 2006; Fleurian et al., 2014), Kolmogorov complexity, e.g., FLAC, Ogg Vorbis, MP3 file compression methods (Marin and Leder, 2013; Fleurian et al., 2014; Güçlütürk, 2018; Güçlütürk et al., 2018), event density (Marin and Leder, 2013), variability of temporal and spectral frequencies (Samson et al., 2011) as well as features extracted by deep neural networks (Güçlü et al., 2016).

Nevertheless, while the concept of musical complexity is not a very well-defined one, it is a rather intuitive one. That being said, this intuition regarding the definition of subjective music complexity could vary among people, for instance, with different levels of music education as suggested by several studies (Orr and Ohlsson, 2001, 2005; Rentfrow et al., 2011; Marin and Leder, 2018) and similar to how subjective categorizations of visual art change with art education (Actis-Grosso et al., 2017).

As suggested by Berlyne (1971), many music studies reported an inverted U-curve relationship between stimulus complexity and liking (North and Hargreaves, 1995; Orr and Ohlsson, 2001). However, other relationships between liking and complexity of music have also been frequently observed (Orr and Ohlsson, 2005; Marin and Leder, 2013; Marin et al., 2016). For instance, Marin et al. (2016) has shown that relationship between complexity and liking followed a positive linear trend, whereas other hedonic dimensions, beauty and pleasantness that they studied resulted in an inverted U-curve and a negative linear relationship, respectively. On the other hand, Orr and Ohlsson (2005) have observed either no relationship between the complexity and liking of music excerpts (evaluated by jazz musicians) or a negative relationship (evaluated by bluegrass musicians).

Besides complexity, factors such as familiarity, emotional valence, music genre (which also relates to complexity), etc. have been known as important modulators of music preferences (for a review see Corrigall and Schellenberg, 2015). In fact, recently Madison and Schiölde (2017) showed that familiarity increases liking independent of the complexity of the music excerpts. Such results highlight the importance of stimulus selection in studies that aim to investigate liking and preferences with ecologically valid stimuli. The songs used in the current study were previously used in several other studies investigating music preferences with robust results (Rentfrow et al., 2011, 2012; Greenberg et al., 2015) and were selected to span a variety of music types and the five factors of the MUSIC model (Rentfrow et al., 2011) allowing us to make more general conclusions rather than e.g., genre specific ones. Furthermore, the songs were selected among not well-known songs in Getty Images to avoid familiarity effects (Rentfrow et al., 2011).

Recently, we demonstrated complexity as a major factor for explaining individual differences in visual preferences for abstract digital art (Güçlütürk et al., 2016). We showed that participants could best be separated into two groups based on their liking ratings for abstract digital art comprising geometric patterns: one group with a preference for complex visual patterns and another group with a preference for simple visual patterns. These two opposite complexity preferences emerged from what initially appeared to be an inverted U-curve when the data were simply averaged. These preference relationships were obtained for a highly-controlled set of visual stimuli, varying in only a few perceptual dimensions such as the number and size of elements.

Our findings were later replicated in the visual domain by identification of robust preference differences for fractal-like images (Spehar et al., 2016), where four different preference patterns were identified: a group with linearly increasing, another group with linearly decreasing, another intermediate group with mid-level spectral slope preference and finally a group without any particular preference. However, the stimuli sets used in this study were also strictly controlled grayscale images, therefore it is still not known whether these results would further generalize to more ecologically valid stimuli and/or other sensory modalities.

In the present study, we extended our investigations for complexity preferences from highly controlled visual stimuli to ecologically valid stimuli in the auditory modality. Our aim was to see if the aggregated analysis of music complexity-liking relationship would misleadingly appear to be different than the actual preference tendencies of clusters of individuals with similar preferences. Additionally, to gain further insights about participant characteristics who have different complexity preferences, we investigated various demographic and personality measures. We tested whether grouping individuals based on differences in their liking ratings for song excerpts would result in a grouping which also reflected distinct complexity preferences. Similar to our previous study in visual preferences (Güçlütürk et al., 2016), here we studied the music preferences and showed how they are related to stimulus complexity. To demonstrate these relations, we used k-means clustering algorithm and cluster a large number of participants based on their liking ratings for song excerpts from various musical genres.

Furthermore, within the limits of the data set we present further *post-hoc* analyses to exemplify how characteristics of individuals with different music complexity preferences in terms of demographic and personality measures can be related to complexity preferences for music.

## 2. Materials and Methods

### 2.1. Data

The data were derived from “Study 2” of Greenberg et al. (2015). This dataset consists of liking ratings by 353 participants for 25 different song excerpts as well as demographic information for the participants, such as age, gender, occupation, etc. and responses to two personality questionnaires measuring (i) emotional quotient (EQ) and (ii) (revised) systemizing quotient (SQ-R). For details regarding the data collection, we refer the reader to Greenberg et al. (2015). Below we briefly describe the relevant aspects.

#### 2.1.1. Participants and Procedures

Participants were Amazon Mechanical Turk workers who participated by filling an online survey that was hosted by Qualtrics. Total number of participants who completed all the required measures was 353. Among these 353 participants, 220 (62%) were female and 133 (38%) were male. The ages of the participants were between 18 and 68 (*M* = 34.10, *SD* = 12.27). This research was given ethical approval by the Psychology Research Ethics Committee of the University of Cambridge in August 2013 in accordance with the Declaration of Helsinki.

#### 2.1.2. Stimuli

Stimulus material consisted of 15-s-long 25 song excerpts that were previously used in several music preference studies (Rentfrow et al., 2011, 2012; Greenberg et al., 2015). The songs were selected by Rentfrow et al. (2011) to span the five broad dimensions of the MUSIC model, which is a model for explaining individual differences in music preferences. MUSIC is an acronym for the following: Mellow (romantic, relaxing, unaggressive, sad, slow, and quiet music; example genres: soft rock, R&B, and adult contemporary); Unpretentious (uncomplicated, relaxing, unaggressive, soft, and acoustic music; example genres: country, folk); Sophisticated (inspiring, intelligent, complex, and dynamic music; example genres: classical, operatic, avant-garde, world beat, and traditional jazz); Intense (distorted, loud, aggressive, and not relaxing, romantic, nor inspiring music; example genres: classic rock, punk, heavy metal, and power pop); and Contemporary (percussive, electric, and not sad music; example genres: rap, electronica, Latin, acid jazz, and Euro pop).

The genres of the 25 stimulus songs were as follows: rock-n-roll, adult contemporary, electronica, soft rock, europop, R&B soul, rap, avant-garde classical, classical, latin, traditional jazz, world beat, classic rock, heavy metal, punk, bluegrass, mainstream country, new country. Since different genres tend to have different instrumental complexity levels (Percino et al., 2014), this variety serves to diversify the levels of complexity in the dataset. A full list of the songs in order of increasing complexity is available in Supplementary Materials.

#### 2.1.3. Measures

In the current study, as part of the demographic measures we used the age and gender variables. Other measures used in the study are described below.

##### 2.1.3.1. Empathy quotient

Empathy was measured using the 60-item self-report EQ questionnaire which measures the affective and cognitive components of empathy in adults (Baron-Cohen and Wheelwright, 2004). Empathy as measured by this questionnaire was defined as follows: “*the drive or ability to attribute mental states to another person/animal, and entails an appropriate affective response in the observer to the other person's mental state*.” Each statement was evaluated by the participants on a four-point scale consisting of: strongly disagree, slightly disagree, slightly agree, or strongly agree.

##### 2.1.3.2. Systemizing quotient-revised

Systemizing was measured using the 75-item SQ-R questionnaire (Wheelwright et al., 2006), and it was defined as follows: “*the drive to analyze, understand, predict, control and construct rule-based systems*.” Similar to EQ, each statement in the questionnaire was evaluated by the participants on a four-point scale consisting of: strongly disagree, slightly disagree, slightly agree, or strongly agree.

##### 2.1.3.3. Brain types

Five cognitive “brain types” as per the E-S theory (Baron-Cohen et al., 2003) were calculated for each participant based on the standardized differences between EQ and SQ measures. The five brain types are defined as follows:

- Type E: Individuals who have more developed empathizing drive/abilities than systemizing ones
- Type B: Individuals who have equally developed empathizing and systemizing drive/abilities
- Type S: Individuals who have more developed systemizing drive/abilities than empathizing ones
- Extreme type E: Individuals who have normal or overdeveloped empathizing drive/abilities and underdeveloped systemizing ones (There were no individuals with this brain type in the current sample)
- Extreme type S: Individuals who have normal or overdeveloped systemizing drive/abilities and underdeveloped empathizing ones

##### 2.1.3.4. Autism spectrum quotient

AQ measures where an individual lies in the continuum of autistic traits (Baron-Cohen et al., 2001). Autism spectrum conditions usually manifest themselves with difficulties in empathy and an increased tendency for systemizing behavior (Wheelwright et al., 2006). In this study, since the original dataset did not contain AQ measurements, AQ for each participant was estimated using their EQ and SQ-R scores as described by Wheelwright et al. (2006). Specifically, AQ was calculated using the following two formulas:

(1) $${\text{AQ}}_{\text{m}}=0.089\text{SQ-R}-0.25\text{EQ}+21.6$$

(2) $${\text{AQ}}_{\text{f}}=0.089\text{SQ-R}-0.25\text{EQ}+22.7$$

for males (AQ_m_) and females (AQ_f_), respectively. In general population, males and females are known to differ in AQ, EQ, and SQ score distributions (Wheelwright et al., 2006; Baron-Cohen, 2010), as reflected by the above formulas.

##### 2.1.3.5. Liking ratings

Each participant listened to all 25 excerpts and provided a rating of how much they liked each excerpt. The ratings were collected on a nine point Likert scale (1 = dislike extremely; 2 = dislike very much; 3 = dislike moderately; 4 = dislike slightly; 5 = neither like nor dislike; 6 = like slightly; 7 = like moderately; 8 = like very much; 9 = like extremely).

##### 2.1.3.6. Complexity ratings

Complexity ratings for the song excerpts were previously collected as part of another study (Rentfrow et al., 2011). In the current study, complexity score of each song is taken as the average complexity rating given by the 40 “judges” that rated the songs in the original study.

##### 2.1.3.7. Complexity preference

Complexity preference of each of the 353 participants were calculated as follows:

(3) $${\text{CP}}^{\left(n\right)}=\sum_{i=1}^{25}{\text{L}}_{i}^{\left(n\right)}{\text{C}}_{i}$$

where CP^(n)^ is the complexity preference of the *n*th participant, L_i_^(n)^ is the liking rating of the *n*th participant for the *i*th song and C_i_ is the complexity score of the *i*th song.

### 2.2. Analyses

Following analyses were performed on the above described data using MATLAB.

**Figure 1:**
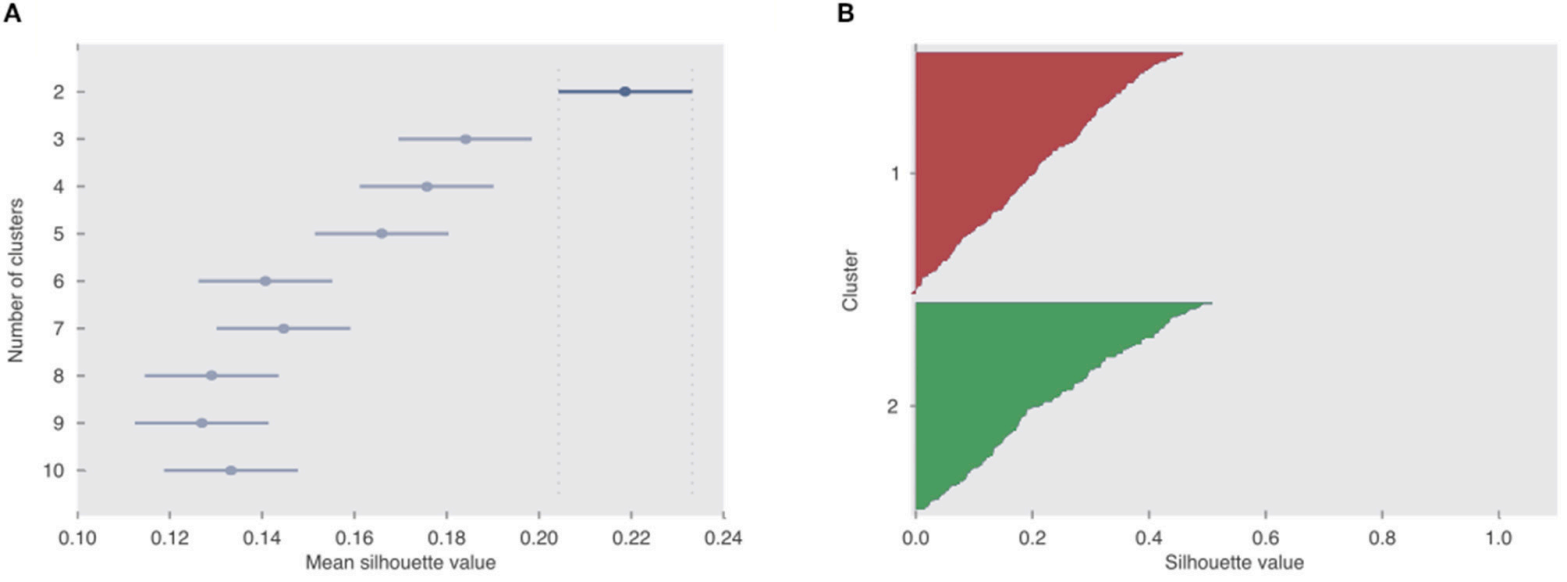
Evaluation of clusters. **(A)** Comparison of average silhouette values of clustering the participants with *k* = [2, 10], where *k* = 2 appears to be a significantly better grouping of the 353 participants compared to the remaining values. **(B)** Silhouette value distribution of participants as they were assigned to Cluster 1 and Cluster 2, where *k* = 2.

**Figure 2:**
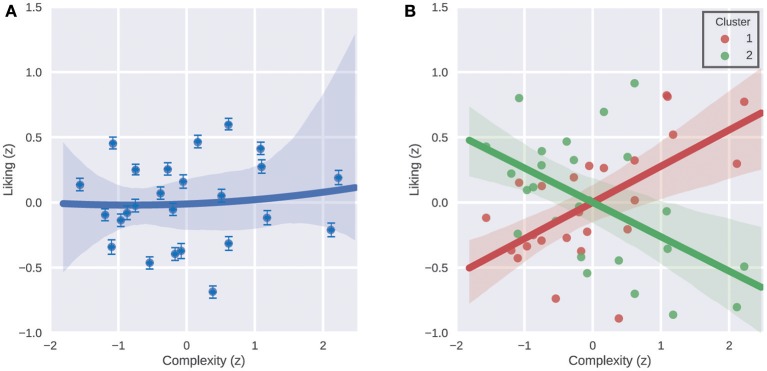
Liking ratings of the participants as a function of stimulus complexity. **(A)** Average normalized liking ratings of all participants vs. normalized complexity scores of the 25 song excerpts. Dots represent the stimuli, and the line represents a quadratic function fit. Shaded area indicates error of fit and the error bars show the standard error of mean. **(B)** Average normalized liking ratings of participants in Cluster 1 and Cluster 2 separately vs. normalized complexity scores of the 25 song excerpts. Dots represent the stimuli, and the two lines represent the regression lines. Shaded area indicates error of fit.

**Figure 3:**
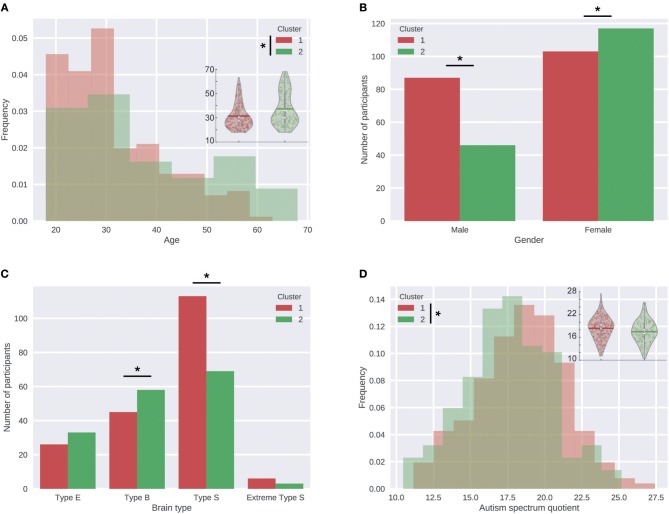
Significant differences between the two clusters. **(A)** Distribution of age in the two clusters. Overlaid violin plots on the right-hand side further detail the age distribution of participants in each cluster. **(B)** Number of participants with each gender in the two clusters. **(C)** Number of participants with each cognitive brain type in the two clusters. **(D)** Distribution of AQ in the two clusters. Overlaid violin plots on the right-hand side further detail the AQ distribution of participants in each cluster. ^*^Indicates significant differences.

1. Participants (*N* = 353) were clustered into 2–10 clusters based on their liking ratings for the 25 songs in the dataset using the k-means clustering algorithm.
2. Silhouette analysis was performed to identify the optimal number of clusters that explain the data. For this analysis a silhouette value for each participant in each cluster assignment was calculated. And then an average silhouette value is calculated for each of the 2–10 sets of clusters (Figure 1). A high average silhouette value indicates good separation between clusters and similarity of elements within the same cluster.
3. Next to the silhouette analysis, intraclass correlation (ICC) indices were calculated for or the whole sample of participants as well as for each cluster to further evaluate the agreement of participants within these groups. A high ICC index represents high agreement.
4. For visual inspection, the data of all participants as well as those assigned to the 2 clusters providing the highest separation were normalized and visualized in terms of Liking vs. Complexity graphs (Figure 2).
5. To statistically compare a single quadratic function and a combination of two linear functions in terms of how much they explain the liking-complexity relationship in the data, we performed regression analyses. We compared two generalized linear mixed models, a quadratic model and a cluster-based model (see Equations 4 and 5 and Table 1 in the results section).
6. The two generalized linear mixed models were compared with a simulated likelihood ratio test with 1,000 simulations (Table 2).
7. In order to characterize the participants assigned to each cluster, summary statistics were calculated for a number of personality and demographic measures (age, gender, brain type, and AQ) in each cluster. These statistics were then compared using the appropriate statistical tests and results were corrected for multiple comparisons with Holm-Bonferroni correction (Table 3).
8. The summary statistics and statistical comparisons were visualized with graphs (Figure 3).
9. Finally, a logistic regression was performed to confirm the effects of age, gender, brain type, and AQ on the assignment of participants to each cluster (Table 4).

**Table 1:** Estimated fixed-effect coefficients of the quadratic and cluster-based models.

**Fixed-effect coefficients**	**Coefficient name**	**Estimate**	**SE**	**DF**	***t*-statistic**	***p*-value**	**Lower CI**	**Upper CI**
Quadratic	Intercept	–0.01	0.01	8822	–0.85	0.4	–0.04	0.02
model	Complexity	0.02	0.01	8822	1.71	0.09	–0.00	0.04
	Complexity^2^	0.01	0.01	8822	1.30	0.2	–0.01	0.03
								
Cluster-	Intercept	~0	0.03	8821	~0	1	–0.06	0.06
based	Complexity	0.82	0.03	8821	25.95	~0	0.76	0.88
model	Cluster	~0	0.02	8821	~0	1	–0.04	0.04
	Complexity × Cluster	–0.54	0.02	8821	–26.48	~0	–0.58	–0.50

*SE, standard error; DF, degrees of freedom; CI, 95% confidence interval*.

**Table 2:** Simulated likelihood ratio test results.

**Model**	**DF**	**AIC**	**BIC**	**Log likelihood**	**LRT- statistic**	***p*-value (CI)**
Quadratic	5	24610	24645	–12300	673.09	0.001
Cluster-based	6	23939	23981	–11963		(0.00003–0.006)

*DF, degrees of freedom; AIC, Akaike information criterion for the model; BIC, Bayesian information criterion for the model; Log Likelihood, maximized log likelihood for the model; LRT-statistic, likelihood ratio test statistic; CI, 95% confidence interval*.

**Table 3:** Characteristics of the participants assigned to the two clusters.

	**All mean ± SD or #participants**	**Cluster 1 mean ± SD or #participants**	**Cluster 2 mean ± SD or #participants**	***p*-value**	**Test statistic (test)**
Age	34.11 ± 12.27	31.36 ± 10.24	37.31 ± 13.63	4 × 10^−5^^**^	χ^2^ = 16.87 (Kruskal-Wallis)
Gender	#F: 220 #M: 133	#F: 103 #M: 87	#F: 117 #M: 46	7 × 10^−4^^*^	χ^2^ = 11.53 (χ^2^)
EQ	41.62 ± 12.37	40.67 ± 12.62	42.74 ± 12.02	0.1	*t* = –1.57 (Student's *t*)
SQ-R	63.90 ± 20.34	65.74 ± 20.84	61.74 ± 19.60	0.07	*t* = –1.85 (Student's *t*)
Brain type	#E: 59 #B: 103#S: 182 #XS: 9	#E: 26 #B: 45 #S: 113 #XS: 6	#E: 33 #B: 58 #S: 69 #XS: 3	0.007^*^	χ^2^ = 12.11 (χ^2^)
AQ_all_	17.98 ± 2.98	18.38 ± 2.96	17.51 ± 2.94	0.003^*^	*t* = 2.77 (Student's *t*)
AQ_f_	17.19 ± 2.86	17.53 ± 2.97	16.88 ± 2.74	0.05	*t* = 1.68 (Student's *t*)
AQ_m_	19.30 ± 2.70	19.40 ± 2.63	19.11 ± 2.84	0.3	*t* = 0.58 (Student's *t*)
CP	0 ± 1^a^	0.68 ± 0.68 (complex)	–0.79 ± 0.68 (simple)	~0^***^^b^	*t* = 20.25(Student's *t*)

* *indicates p-values < 0.05*,

** indicates p-values < 0.001, and

*** *indicates p-values < < 0.001 after Holm-Bonferroni correction*.

a *Note that complexity preference was normalized to have zero mean and unit variance*.

b *Note that since the normalized complexity preference is defined as a function of liking ratings, it is expected to be significantly different in the two clusters*.

**Table 4:** Results of logistic regression with age, gender, brain type, and AQ variables.

	**β**	**SE**	**DF**	***p*-value**	**odds ratio**	**Lower CI**	**Upper CI**
Age	0.04	0.01	1	0.000	1.04	1.02	1.06
Gender(1)	0.57	0.25	1	0.022	1.77	1.08	2.89
AQ	0.00	0.08	1	0.998	0.99	0.85	1.17
Brain type	–	–	3	0.565	0.57	–	–
Brain type(1)	–0.20	0.86	1	0.817	0.82	0.15	4.39
Brain type(2)	0.50	0.59	1	0.400	1.65	0.51	5.27
Brain type(3)	0.52	0.37	1	0.160	1.68	0.81	3.48
Constant	–2.12	1.64	1	0.198	0.12	–	–

*SE, standard error; DF, degrees of freedom; CI, 95% confidence interval for odds ratio (expβ)*.

## 3. Results

### 3.1. Clusters

Participants were clustered into groups based on their liking ratings for the 25 songs. For this, k-means clustering algorithm (Lloyd, 1982) was used. To explain briefly, cluster analysis groups individual elements in a set such that the similarity of elements assigned to the same cluster is maximized, whereas the similarity between different clusters is minimized. A distance measure between the elements is often used as a measure of similarity. K-means clustering algorithm iteratively partitions data into *k* clusters by assigning each data point to a cluster such that the within cluster sum of squares is minimized. This is equivalent to assigning each data point to the nearest cluster.

Here, k-means clustering algorithm was used for clustering the 353 participants into subsamples such that participants with similar song preferences would be assigned to the same cluster. Specifically, we clustered the participants based on their liking ratings of the 25 songs. We tested several numbers of clusters (*k* = [2, 10]) to determine the best separation of the participants (Figure 1). After obtaining the clusters, to determine the optimum number of clusters, average silhouette values across all data were calculated for each value of k. The silhouette value measures the within cluster similarity of a data point in comparison to its between cluster similarity (Rousseeuw, 1987). In other words, a high average silhouette value indicates good separation between different clusters as well as showing that the similarity of elements within the same cluster is high. We found that when *k* = 2, the average silhouette value was significantly larger than the remaining values of *k* (Student's *t*-test, *p* < 0.001 for all comparisons, Bonferroni corrected for multiple comparisons). Since a high average silhouette value indicates a better clustering, we concluded that clustering participants into two groups resulted in the best separation and the most appropriate grouping of the participants (Rousseeuw, 1987). Therefore, further analyses were performed on these two clusters. Specifically, in Cluster 1 there were 190 participants (103 females and 87 males, average age ± SD = 31.36 ± 10.24) and in Cluster 2 there were 163 participants (117 females and 46 males, average age ± SD = 37.31 ± 13.63).

Additionally, we calculated the intraclass correlation (ICC) indices for the whole sample of participants as well as for each cluster to evaluate the agreement of participants within these groups (Shrout and Fleiss, 1979). Specifically, we calculated ICC(2, 1) i.e., two-way random single measures for all the participants together, for participants in Cluster 1 alone, and for those in Cluster 2 alone. ICC(2, 1) for all participants was 0.10, whereas after clustering the participants it increased to 0.20 and 0.24 for Cluster 1 and Cluster 2, respectively. Clustering the participants significantly increased the ICC indices (*F*-test, *p* < 0.001). This analysis confirms that clustering the participants allowed us to obtain subsets of the sample that agreed more with each other.

We then calculated the average normalized liking ratings of all participants and plotted these average ratings vs. normalized complexity scores of the 25 song excerpts. We did the same for the ratings of the participants who were assigned to Cluster 1 and Cluster 2 separately as well (Figure 2). Visual inspection of these liking vs. complexity graphs of the two clusters revealed clearly distinct relationships between the liking and complexity variables. While the 190 participants in Cluster 1 on average had a clear preference for more complex songs, the 163 participants in Cluster 2 had an opposite average preference pattern of a preference for simpler songs.

Next, to statistically compare a single quadratic function and a combination of two linear functions in terms of how much they explain the liking-complexity relationship in the data, we performed regression analyses. We compared the following two generalized linear mixed models:

(4) $$\begin{array}{l} \text{Liking}=\beta_{0}+\beta_{1}\text{Complexity}+\beta_{2}{\text{Complexity}}^{2}\\ \;+b_{0}\text{Participant}+\epsilon \end{array}$$

(5) $$\begin{array}{l} \text{Liking}=\beta_{0}+\beta_{1}\text{Complexity}+\beta_{2}\text{Cluster}+\\ \;\beta_{3}\text{Complexity}\times \text{Cluster}+b_{0}\text{Participant}+\epsilon \end{array}$$

where β_*i*_ denotes the fixed-effect coefficients, *b*_0_ denotes the random-effect coefficients, and ϵ denotes the residuals. Random-effects terms were included in both models in order to account for the repeated measures structure of the data. Models were implemented using MATLAB. Since liking ratings were observed to be normally distributed, normally distributed responses and identity link function options were selected during the implementation. Table 1 shows the estimated coefficients of the two models.

Next, the two models were compared using a simulated likelihood ratio test with 1,000 simulations. Table 2 shows the results of simulated likelihood ratio test. The cluster-based model had lower Akaike information criterion (AIC) value and Bayesian information criterion (BIC) value than the quadratic model, indicating that the cluster-based model is the better fitting model (Hox et al., 2010). Note that the *p*-value for the simulated likelihood ratio test was less than 0.001, further demonstrating that the cluster based model significantly better explains the data.

In summary, these analyses demonstrate that rather than averaging the data of all participants, grouping them by means of a clustering algorithm results in two groups with more homogenous and distinct complexity preference patterns that better explain the data. Furthermore, these two opposite complexity preference patterns that we now show for music stimuli coincide with the earlier findings that were established in the visual modality (Güçlütürk et al., 2016). Taken together, these results suggest that this complexity-liking relation is not restricted to a specific domain and that it could rather be a supramodal characteristic of sensory preferences.

### 3.2. Characterizing the Participants in the Different Clusters

Next, we performed a number of *post-hoc* analyses to investigate the characteristics of the people assigned to different clusters. Here we were obviously bound to the specific characteristics measured in the original study (Greenberg et al., 2015).

As demographic measures, we looked at whether age and gender distribution differed between the two clusters. As personality measures, we looked at the distributions of EQ, SQ, “brain type,” and AQ, which we estimated using EQ and SQ scores of individuals. We will first focus on each of the demographic and personality measures, and next consider their mutual contribution to determine the relative influence of these characteristics (by applying a logistics regression analyses).

Table 2 shows the how these characteristics were among the whole sample and the clusters as well as the results of Holm-Bonferroni corrected statistical tests comparing the two clusters. We found that both demographic and personality measures were informative for characterizing the participants in different clusters (Figure 3 and Table 3). Particularly, we found that participants in Cluster 2 who preferred simpler music were significantly older than those in Cluster 1 who preferred more complex songs. In terms of the gender distribution, majority of the male participants were assigned to Cluster 1 (65% of the males were assigned to Cluster 1 and 35% to Cluster 2), whereas for females this pattern was reversed and the difference between the number of female participants in the two clusters was smaller (47% of the females were assigned to Cluster 1 and 53% to Cluster 2). While tests showed that the gender and age had a significantly different distribution in the two clusters, the distribution of the personality predictors EQ and SQ did not differ. However, we found significant differences between the distribution of different brain types (especially type S and type B) in the two clusters. Specifically, those participants who were type S were more likely to be assigned to the cluster that preferred complex stimuli (i.e., Cluster 1), whereas the participants with type B brain types were more likely to prefer simple stimuli (i.e., be assigned to Cluster 2).

Based on the literature that identified enhanced sensory processing in autism spectrum condition (ASC) (Dakin and Frith, 2005; Haesen et al., 2011) in combination with the fluency theory of aesthetic pleasure (Reber et al., 2004), we hypothesized that the participants who were assigned to the cluster with high complexity preference, i.e., Cluster 1, would on average have higher AQ levels. As expected, one-tailed Student's *t*-tests comparing the two clusters revealed that the average AQ was significantly higher for the participants of Cluster 1 compared to Cluster 2 (*p* = 0.003). However, when controlled for gender, this difference lost its statistical significance (*p* = 0.0475, did not survive Holm-Bonferroni correction for multiple comparisons).

Next, a logistic regression was performed to confirm the effects of age, gender, brain type and AQ on the assignment of participants to each cluster (Table 4). The logistic regression model was statistically significant, χ^2^ (6) = 36.55, *p* < 0.001. The model explained 13% (Nagelkerke *R*^2^) of the variance in the cluster assignment and correctly classified 64% of cases, indicating that there are other unaccounted factors influencing the cluster assignment. Age (β = 0.04, *p* < 0.001) and gender (β = 0.57, *p* = 0.02) added significantly to the model, whereas brain types (*p* = 0.57) and AQ (*p* = 0.99) did not have a significant effect. According to the model, males were 1.77 times more likely to be assigned to the Cluster 1 (high complexity preference) than females. Furthermore, increasing age was associated with an increased likelihood of assignment to Cluster 2 (low complexity preference).

## 4. Discussion

In this study, we showed that the previously observed duality of complexity preferences (Güçlütürk et al., 2016) was not only limited to the visual domain and highly controlled stimuli, but also was evident in the auditory domain with complex ecologically valid music stimuli. We demonstrated the importance of accounting for individual differences by revealing opposite complexity preference patterns for two groups of participants in a sample of over 300 participants. This result resembles our previous study in the visual domain in which the same analyses on the appreciation of visual stimuli led to the decomposition of the inverted U-curve into two subgroups of participants (complexity likers and complexity dislikers, Güçlütürk et al., 2016).

Furthermore, to exemplify how personality characteristics can be related to one of the subgroups, we presented *post-hoc* analyses to characterize the identified groups with different complexity preferences. Remarkably, similar to previous reports regarding music preferences (North, 2010), the results of our analyses suggest that demographic measures were the most important variables predicting complexity preferences. Specifically, we found that younger people were more likely to prefer complex songs whereas for older people it was the opposite. Furthermore, males were more likely to be assigned to the group with high complexity preference, whereas females were almost equally distributed in the two clusters, but they were slightly more likely to be assigned to the cluster with low complexity preference. Here, caution for overgeneralization is needed.

The result that grouping the participants increased the ICC as well as improving the fit of the linear mixed models demonstrates the necessity of using simple yet effective methods like clustering for evaluating the effects of modulating factors of liking. Our results show that separating this relatively large sample of participants into two groups reveals the best grouping, and on average these two groups have opposite complexity preferences. However, it is important to be aware of the further variability within these two groups that may be driven by factors other than complexity. Although the two opposite liking vs. complexity functions are evident even upon visual inspection in Figure 2 (as well as quantitatively in Tables 1, 2), the linear relationships between liking and complexity in the clusters are still noisy. We believe that such variability in preferences is expected as the stimulus songs varied in the many dimensions of the MUSIC model (Rentfrow et al., 2011) and were not controlled in many aspects. Besides introducing some level of noise/variability to the results, another consequence of using such an uncontrolled (and ecologically valid) stimulus set is that it allows making general conclusions spanning the large extent of the music domain. While the current results provide evidence for a general relation between complexity and liking in music, investigating within genre preferences with similar methods would be an interesting next step in the study of complexity preferences for music.

With respect to the characterization of participants with simple and complex music preferences, the observed age differences between the two clusters of participants is an interesting but not an unexpected result. Very recently, Pugach et al. (2017) showed that visual aesthetic preferences are not stable across the lifespan. Our results conform to this finding and suggest that older participants were more likely to prefer simpler songs whereas the younger participants were more likely to prefer more complex songs. In the visual domain, such a relationship has been suggested earlier (Munsinger et al., 1964; Alpaugh and Birren, 1977; Crosson and Robertson-Tchabo, 1983; Güçlütürk et al., 2016). In the auditory domain, an important role of age in music preferences has been established (Bonneville-Roussy et al., 2013, 2017), however these investigations were more focused on music genres rather than the complexity dimension of music. Although it is difficult to disentangle the impact of complexity on the perception and categorization of musical genres, it is important to also consider possible effects of age-related differences in genre preferences. There are only a few studies that investigated gender differences in musical complexity preferences (Marin and Leder, 2013), Previously, Marin and Leder (2013) found a significant positive correlation between complexity and pleasantness of music pieces (rs = 0.41) for male participants, and a negative but not significant relationship for female participants (rs = –0.14). When controlled for the effects of familiarity, they found that the relationship between arousal and pleasantness got stronger only for males (rs = 0.35) but not for females (rs = –0.23). Therefore, they suggested that the relationship between complexity and pleasantness cannot be meaningfully discussed without considering gender. The results of the current study are in line with these results, and thus emphasizes gender as an important factor. Future studies should further investigate its role in complexity preferences for art and music.

Our initial analysis showed that there were small but significant differences between the two clusters in terms of their average AQ, such that people that were assigned to the high complexity preference cluster had a significantly higher average AQ. However, the results of the logistic regression analysis suggest that this difference was likely driven by the gender differences. Since the results of the logistic regression analysis did not reveal a significant relationship between the brain types and the complexity preferences in contrast with our initial tests, we avoid extensive discussions on these initial results. Nevertheless, it would be interesting to investigate a possible link between superior or abnormal sensory processing as in ASC (Dakin and Frith, 2005; Haesen et al., 2011; Robertson and Simmons, 2012) and preferences to better explain the current results in connection to recent findings regarding strong links between visual sensitivity and preferences (Spehar et al., 2015).

With regard to the *post-hoc* analyses on personality characteristics and demographic variables a warrant is in place. It should be noted, that the current specific results on age and gender are basically restricted to this particular data set. Many more participant characteristics can potentially be measured and related to the different tendencies as revealed by the decomposition of the inverted U-curve. Future studies should further investigate the role of such variables in complexity preferences also using different music styles. Here the important finding is that our earlier proposed clustering method for complexity-liking data regarding the appreciation of visual patterns appears to reveal similar results in the music domain, which again decomposes the initial inverted U-curve and breaks down the overall pattern in participant-related subsets of data.

We believe that the study of aesthetics can benefit from simple yet effective approaches that we illustrated in the current study. Indeed, currently more frequently in the visual domain, increasingly higher number of reports (see e.g., the recent studies by Mallon et al. 2014; Spehar et al. 2016; Viengkham and Spehar 2018; Muth et al. 2018), which study and demonstrate differences in preferences and art perception via clustering approaches, illustrate the utility of this approach. Pooling preference data across participants may easily obscure relevant differential tendencies between groups of participants. Diving into the nature of these differences is likely to reveal new insights in the underlying mechanisms and inter-individual differences driving these data.

On top of the above mentioned future research directions, the approach and the results of the current study generate several new research questions as listed below.

- Is the duality of complexity preferences limited to simple visual patterns and music of various genres or can it be observed with other sets of stimuli, for example within a specific genre of songs, or a varied set of paintings? To what extent do these results generalize?
- Does the preference in a modality also depend on sensory sensitivity, and if so, what type of sensitivity could potentially account for these differences?
- Does the complexity preference in one modality also persist in the other modality, i.e., if a person likes complex music, would they also like complex visual art? If this is the case, this may suggest a different supramodal mechanism and an explanation other than sensory sensitivity. Such results would further necessitate moving toward neuroaesthetics theories encompassing different sensory modalities (Marin, 2015; Brattico et al., 2017).

## Author Contributions

YG and RvL designed the research. YG obtained and analyzed the data. YG and RvL interpreted the results and wrote the manuscript.

### Conflict of Interest Statement

The authors declare that the research was conducted in the absence of any commercial or financial relationships that could be construed as a potential conflict of interest.

## Footnotes

^1^According to Shannon's information theory, messages that have a high probability of occurrence, have less information content (and are less complex) compared to low probability messages (which are more complex).
