# Supplementary material for: Decomposing Complexity Preferences for Music
Source: Front Psychol. 2019 Apr 3;10:674. doi: 10.3389/fpsyg.2019.00674 (PMC6457315; doi:10.3389/fpsyg.2019.00674)
Supplement: Supplementary file 1 [file Data_Sheet_1.PDF]

Supplementary Material:
Decomposing complexity preferences for music

1 SUPPLEMENTARY MATERIALS

Table S1. List of songs in the order of increasing complexity

	Artist	Song	Genre
**q5**	Human Signals	Birth	soft rock
**q4**	Language Room	She Walks	soft rock
**q25**	Ali Handal	Sweet Scene	soft rock
**q1**	Lisa McCormick	Let's Love	adult contemporary
**q23**	Bob Delevante	Penny Black	new country
**q11**	Ljova	Seltzer, do I drink too much	avant-garde classical
**q24**	Curtis	Carrots and Grapes	rock-n-roll
**q3**	Kush	Sweet 5	electronica
**q22**	Carey Sims	Praying for Time	mainstream country
**q2**	Leo The Lionheart	050107 electro	electronica
**q15**	Moh Alileche	North Africa's Destiny	world beat
**q6**	AB+	Recess	electronica
**q10**	Ciph	Brooklyn Swagger	rap
**q13**	Lisa McCormick	Fernando Esta Feliz	latin
**q21**	Anglea Motter	Mama I'm Afraid To	bluegrass
**q14**	Daniel Nahmod	I Was Wrong	traditional jazz
**q12**	DNA	La Wally	classical
**q7**	The Cruxshadows	Go Away	europop
**q16**	Exit 303	Falling Down 2	classic rock
**q17**	Cougars	Dick Dater	classic rock
**q19**	The Stand In	Frequency of a Heartbeat	punk
**q18**	Five Finger Death Punch	White Knuckles	heavy metal
**q20**	Straight Outta Junior High	Over now	punk
